# Favorable efficacy of rituximab in ANCA-associated vasculitis patients with excessive B cell differentiation

**DOI:** 10.1186/s13075-020-02215-x

**Published:** 2020-06-15

**Authors:** Yusuke Miyazaki, Shingo Nakayamada, Satoshi Kubo, Yuichi Ishikawa, Maiko Yoshikawa, Kei Sakata, Shigeru Iwata, Ippei Miyagawa, Kazuhisa Nakano, Yoshiya Tanaka

**Affiliations:** 1 School of Medicine, University of Occupational & Environmental Health, 1-1 Iseigaoka, Yahata-nishi, Kitakyushu, Fukuoka 807-8555 Japan; 2 Mitsubishi Tanabe Pharma, Yokohama, Japan

**Keywords:** ANCA-associated vasculitis, B cell, Rituximab

## Abstract

**Objectives:**

B cell depletion by rituximab (RTX) is an effective treatment for anti-neutrophil cytoplasmic autoantibody (ANCA)-associated vasculitis (AAV). However, peripheral B cell phenotypes and the selection criteria for RTX therapy in AAV remain unclear.

**Methods:**

Phenotypic characterization of circulating B cells was performed by 8-color flow cytometric analysis in 54 newly diagnosed AAV patients (20 granulomatosis with polyangiitis and 34 microscopic polyangiitis). Patients were considered eligible to receive intravenous cyclophosphamide pulse (IV-CY) or RTX. All patients also received high-dose glucocorticoids (GC). We assessed circulating B cell phenotypes and evaluated the efficacy after 6 months of treatment.

**Results:**

There were no significant differences in the rate of clinical improvement, relapses, or serious adverse events between patients receiving RTX and IV-CY. The rate of Birmingham Vasculitis Activity Score (BVAS) improvement at 6 months tended to be higher in the RTX group than in the IV-CY group. The proportion of effector or class-switched memory B cells increased in 24 out of 54 patients (44%). The proportions of peripheral T and B cell phenotypes did not correlate with BVAS at baseline. However, among peripheral B cells, the proportion of class-switched memory B cells negatively correlated with the rate of improvement in BVAS at 6 months after treatment initiation (*r* = − 0.28, *p* = 0.04). Patients with excessive B cell differentiation were defined as those in whom the proportion of class-switched memory B cells or IgD^−^CD27^−^ B cells among all B cells was > 2 SDs higher than the mean in the HCs. The rate of BVAS remission in patients with excessive B cell differentiation was significantly lower than that in patients without. In patients with excessive B cell differentiation, the survival rate, the rate of BVAS-remission, and dose reduction of GC were significantly improved in the RTX group compared to those in the IV-CY group after 6 months of treatment.

**Conclusions:**

The presence of excessive B cell differentiation was associated with treatment resistance. However, in patients with circulating B cell abnormality, RTX was effective and increased survival compared to IV-CY. The results suggest that multi-color flow cytometry may be useful to determine the selection criteria for RTX therapy in AAV patients.

## Introduction

Anti-neutrophil cytoplasmic autoantibody (ANCA)-associated vasculitis (AAV) is a systemic autoimmune disease with poor prognosis that damages various organs and commonly occurs in the elderly [1]. To induce remission, AAV is treated with high-dose glucocorticoid (GC) therapy combined with cyclophosphamide (CY), azathioprine (AZA), etc. [2]. However, because these immunosuppressants are nonspecific and cause adverse reactions, development of therapeutic agents specific to the pathology of this disease is warranted. The efficacy of B cell depletion therapy has been demonstrated in AAV patients by the Rituximab in ANCA-Associated Vasculitis (RAVE) trial [3] and the Randomized Trial of Rituximab Versus Cyclophosphamide for ANCA-Associated Renal Vasculitis (RITUXVAS) [4] trial. Rituximab (RTX) has been shown to be as effective as CY for inducing remission in AAV patients. However, it is unknown how B cells actually affect disease activity and prognosis in AAV patients. Furthermore, although RTX, which is the first biologic drug approved for the treatment of AAV, has a specific action mechanism of depleting B cells, the differential use of RTX and CY remains a major clinical question.

The surface phenotypes of peripheral lymphocytes in patients with autoimmune disease are often considered to reflect affected tissue states. We have analyzed peripheral lymphocytes from patients with systemic lupus erythematosus (SLE) [5], rheumatoid arthritis [6], and psoriatic arthritis [7], using 8-color flow cytometry. In these studies, we showed that analysis of peripheral lymphocytes provides valuable insight into the lineage, differentiation stage, activation state, etc., of lymphocytes associated with pathological conditions and can be an indicator for treatment selection.

Thus, the present study aimed to identify abnormalities in the differentiation of peripheral lymphocytes in AAV patients, analyze the clinical significance of these abnormalities, and investigate how to differentiate the use of RTX and CY according to abnormalities in lymphocytes.

## Materials and methods

### Patients

The present study included 54 patients with AAV newly diagnosed by the Watts algorithm [8] who were admitted to our hospital for progressive organ dysfunction (e.g., the lung, kidney, and nervous system) and received high-dose GC therapy between March 2013 and June 2018. Of these patients, remission induction therapy was instituted with RTX in 34 patients and with intravenous CY pulse in 20 patients. The drugs used for remission induction therapy were selected by attending physicians who took baseline patient characteristics into account but remained blinded to B cell phenotypes. The treatment regimens are shown in Supplementary Method. Moreover, healthy individuals matched for age and sex were included as healthy controls (HCs). The Human Ethics Review Committee of University of Occupational & Environmental Health, Japan, reviewed and approved this study, including the collection of peripheral blood samples from HCs and AAV patients. Each subject provided a signed consent form.

### Treatments

All patients received high-dose glucocorticoid therapy (prednisolone 1 mg/kg/day) for 4 weeks. The rituximab group received intravenous rituximab (at a dose of 375 mg per square meter of body-surface area once weekly for 4 weeks). The IV-CY group received intravenous cyclophosphamide (at a dose of 15 mg/kg at 2-week intervals for 12 weeks). Glucocorticoid dosage was reduced to 10 to 20% per 1 to 2 weeks from 4 weeks to 6 months while accommodating individual patient response.

### Clinical measurements

Disease activity was assessed by the Birmingham Vasculitis Activity Score (BVAS) 2003 [9] at baseline and 6 months of treatment. BVAS remission defined as BVAS = 0. The rate of BVAS improvement was {(BVAS at baseline − BVAS at 6 months of treatment)/BVAS at baseline)} × 100(%)).

### Flow cytometric analysis

All patients in this study were enrolled in the FLOW registry, with immunophenotyping analysis performed by multicolor flow cytometry, and the results were recorded in the registry. Peripheral blood (PB) mononuclear cells (PBMCs) were isolated from PB samples using lymphocyte separation medium (ICN/Cappel). Next, PBMCs were resuspended in phosphate-buffered saline (PBS)/3% human IgG (Baxter) in order to block Fc receptors and prevent nonspecific antibody binding, followed by incubation for 15 min at 48 °C in the dark. The cells were then washed with PBS containing 1% bovine serum albumin. Background fluorescence was assessed using appropriate isotype- and fluorochrome-matched control monoclonal antibodies. After staining with antibodies, the cells were assessed by multicolor flow cytometry (FACS Verse; BD Biosciences), and the data were analyzed with FlowJo software (Tree Star). Phenotypes of immune cell subsets were defined based on the Human Immunology Project protocol for comprehensive 8-color flow cytometric analysis proposed by the National Institutes of Health/Federation of Clinical Immunology Societies. Details of the procedure of flow cytometric analysis and gating strategy are shown in Supplementary Table S1 and Fig. 4.

### Statistical analysis

Continuous data are expressed as the mean ± standard deviation (SD) and median (range), and categorical data are expressed as *n* (%). The significance of differences between groups was assessed by Student’s *t* tests and Mann–Whitney *U* tests, with Fisher’s exact test used for nominal variables. When comparing among four categories, statistical significance was determined by *p* < 0.05/4 using Bonferroni method to avoid multiplicity. The Kaplan–Meier method was used to assess the survival rates and the differences were analyzed by the log-rank test.

## Results

### Patient backgrounds

The mean age of all patients was 70.6 ± 8.3 years, and there were roughly the same number of men and women (male to female = 54:46). All patients had new-onset disease. Microscopic polyangiitis was diagnosed in 34 patients (63.0%) and granulomatosis with polyangiitis in 20 patients (37.0%). For remission induction therapy, the patients were divided into those receiving high-dose GC therapy combined with RTX (RTX group) and those receiving GC therapy combined with intravenous CY pulse (IV-CY group). The baseline patient characteristics in these groups are compared in Table 1. In both groups, the common organ dysfunctions were respiratory dysfunction and nephropathy. BVAS was 17.4 ± 6.5 in the RTX group and 15.1 ± 4.6 in the IV-CY group, showing that disease activity was high in the majority of the patients. Between the RTX and IV-CY groups, no significant differences were observed in age, sex, disease duration, vasculitis type, BVAS, organ dysfunction, or GC dose. Concomitant AZA treatment tended to be used more frequently in the IV-CY group than in the RTX group.

**Table 1:** Baseline AAV patient characteristics by treatment group

Variables	Rituximab group, *n* = 34	IV-CY group, *n* = 20	*p* value
Age (years)	70.4 (8.4)	70.9 (8.4)	0.86
Gender, *n* (% female)	19 (55.9%)	10 (50.0%)	0.78
Disease duration (month)	3 (2–5)	4 (1–7)	0.93
New onset, *n* (%)	34 (100.0%)	20 (100.0%)	1.00
ANCA-positive at diagnosis, *n* (%)			
Proteinase 3 ANCA	4 (11.8%)	0 (0.0%)	0.28
Myeloperoxidase-ANCA	26 (76.5%)	18 (90.0%)	0.29
Proteinase 3 + myeloperoxidase-ANCA	3 (8.8%)	1 (5.0%)	1.00
ANCA-associated vasculitis type, *n* (%)			0.24
MPA	19 (55.9%)	15 (75.0%)	
GPA	15 (44.1%)	5 (25.0%)	
BVAS	17.4 (6.5)	15.1 (4.6)	0.15
Organ involvement, *n*			
Cutaneous involvement	2	0	0.52
Mucous membranes and eyes	3	2	1.00
Ear, nose, and throat	11	6	1.00
Pulmonary involvement	27	15	0.74
Renal involvement	28	18	0.75
Neurologic involvement	11	3	0.21
GC dose at base line	56.3 (11.5)	61.6 (16.7)	0.17

Values listed as mean (SD) and median (minimum-maximum) unless otherwise stated. The statistical difference was determined by Student’s *t* test and Mann-Whitney’s *U* test, with chi-square test used for nominal variables. Difference with *p* < 0.05 was considered significant

### No difference in the rate of remission in BVAS between groups

**Fig. 1:**
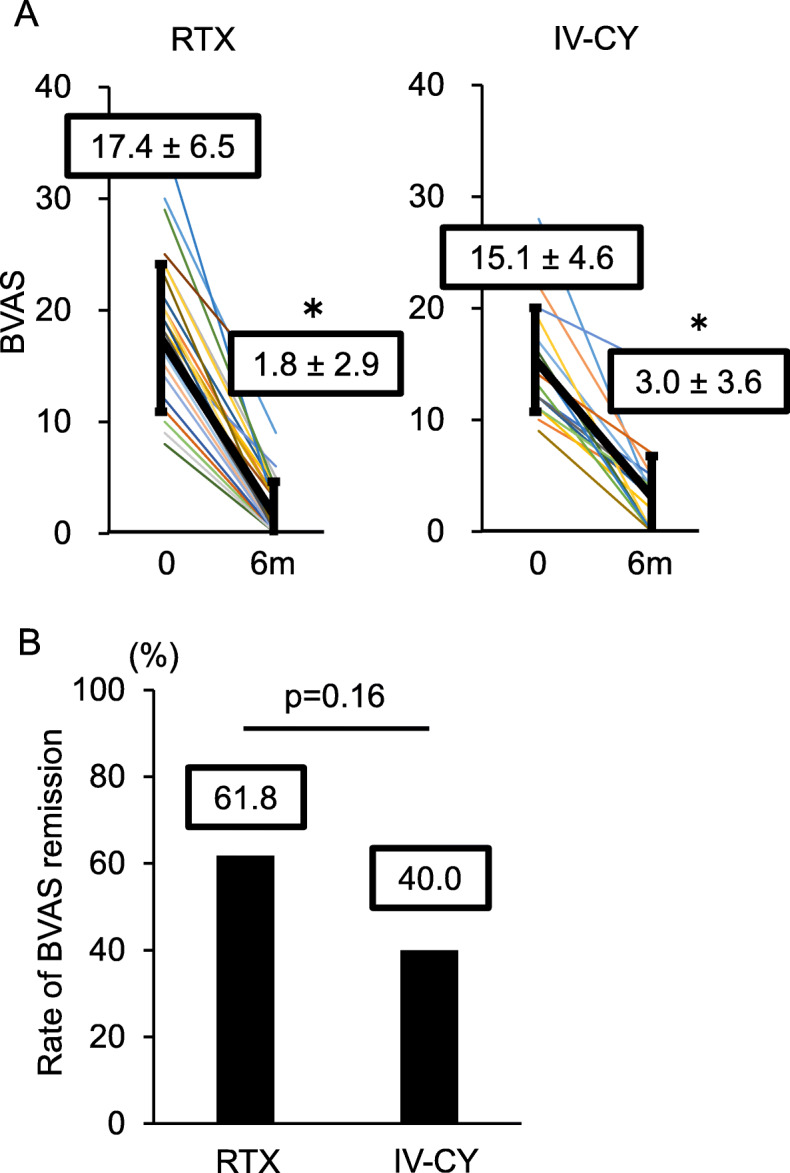
Changes in disease activity after 6 months. **a** Changes in BVAS after 6 months of induction therapy by treatment group. Data are mean ± SD of BVAS in each treatment group. *p < 0.01 according to paired Student’s *t* test. **b** Rate of remission in BVAS by treatment group. Statistical differences were determined by Fisher’s exact test

First, the therapeutic effects of RTX and conventional therapy on AAV were assessed. At 6 months after treatment initiation, BVAS was significantly improved in both the RTX and IV-CY groups (mean change: − 14.2 ± 6.3, *p* < 0.01 in the RTX group; − 15.2 ± 5.1, *p* < 0.01 in the IV-CY group) (Fig. 1a). The rate of remission in BVAS at 6 months after treatment initiation tended to be higher in the RTX group than in the IV-CY group, but this difference was not statistically significant [RTX group = 61.8%, IV-CY group = 40.0%, *p* = 0.16] (Fig. 1b). In addition, no significant differences in the incidences of adverse events, severe infection, leukopenia, or thrombocytopenia or in mortality were observed between the two groups (Table 2). The most common adverse event was infection in both groups, and most deaths were caused by severe infection.

**Table 2:** Adverse events by treatment group

Variables	Rituximab group, *n* = 34	IV-CY group, *n* = 20	*p* value
All adverse events, no.	19	14	0.39
Death, no	4	4	0.45
	Sepsis (pyelonephritis)	Bacterial pneumonia	
	Invasive pulmonary aspergillosis	PCP + invasive pulmonary aspergillosis	
	Bacterial pneumonia	Invasive pulmonary aspergillosis	
	Stroke	Sepsis (bacterial pneumonia)	
Leukopenia, no	4	4	0.45
Thrombocytopenia, no	4	2	1.00
Severe infection, no	11	12	0.09
	CMV infection, *n* = 2	PCP	
	Bacterial pneumonia, *n* = 2	CMV infection, *n* = 2	
	Legionella pneumonia	Acute cholangitis	
	Sepsis (pyelonephritis)	Tuberculosis	
	Invasive pulmonary aspergillosis	PCP + invasive pulmonary aspergillosis	
	PCP	Bacterial pneumonia, *n* = 4	
		Invasive pulmonary aspergillosis	
		Sepsis (bacterial pneumonia)	
VTE, no	1	0	0.37

The statistical difference was determined by chi-square test. Difference with *p* < 0.05 was considered significant

*PCP* pneumocystis pneumonia, *CBV* cytomegalovirus, *VTE* venous thromboembolism

### Increased proportion of IgD^−^CD27^−^ double-negative memory B cells in patients with AAV

**Fig. 2:**
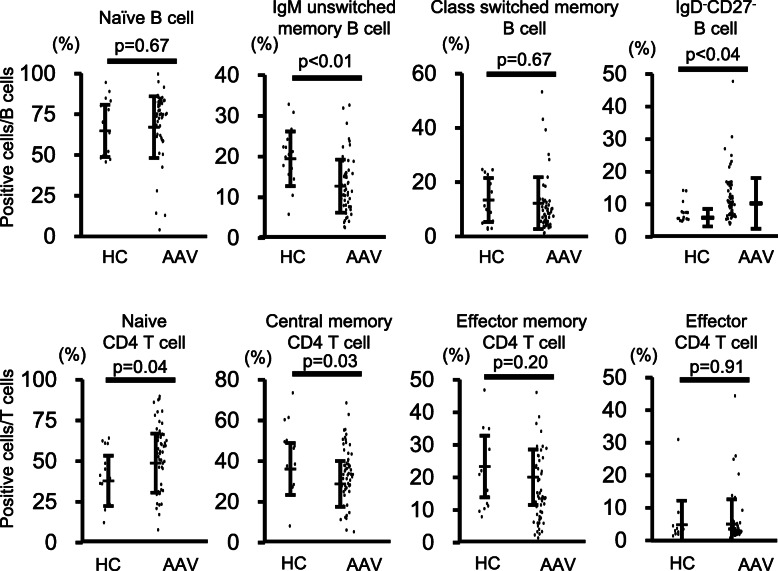
Proportions of peripheral T and B cell phenotypes in healthy control subjects and AAV patients at baseline. The statistical difference was determined by Student’s *t* test. Difference with *p* < 0.05 was considered significant. The horizontal bar represents the mean value. Error bars represent the standard deviation

Phenotypes of peripheral T and B cells before treatment initiation were analyzed by 8-color flow cytometry and compared between AAV patients and 15 HCs matched for age and sex (Fig. 2, Supplementary Table S2). Among cluster of differentiation (CD)-4 T cells, the proportion of naïve CD4 T cells was significantly higher in AAV patients than in HCs, whereas the proportion of central memory CD4 T cells was significantly lower. As for CD8 T cells, no differences in phenotype were observed between AAV patients and HCs. By contrast, in B cells, the proportion of immunoglobulin (Ig)-M unswitched memory B cells was significantly lower in AAV patients than in HCs (HC = 19.0 ± 6.9, AAV = 12.1 ± 6.7, *p* < 0.01), whereas the proportion of IgD^−^CD27^−^ double-negative B cells was significantly higher (HC = 5.4 ± 2.7, AAV = 9.8 ± 7.9, *p* = 0.04]. The absolute number of IgM+ unswitched memory B cells was significantly smaller in patients with AAV than in healthy controls (HC to AAV = 16.8 ± 7.5/μl:7.6 ± 7.9/μl, *p* < 0.001). On the other hand, the absolute number of double-negative B cells did not differ significantly between healthy controls and patients with AAV (HC to AAV = 4.9 ± 3.0/μl:6.0 ± 5.8/μl, *p* = 0.48). These data are shown in Supplementary Fig. 2. There was a positive correlation between the proportion of class-switched memory B cells and the proportion of IgD^−^CD27^−^ B cells in patients with AAV (*n* = 54), as shown in Supplementary Fig. 3 (*r* = 0.50, *p* < 0.001, Pearson product-moment correlation coefficient).

**Fig. 3:**
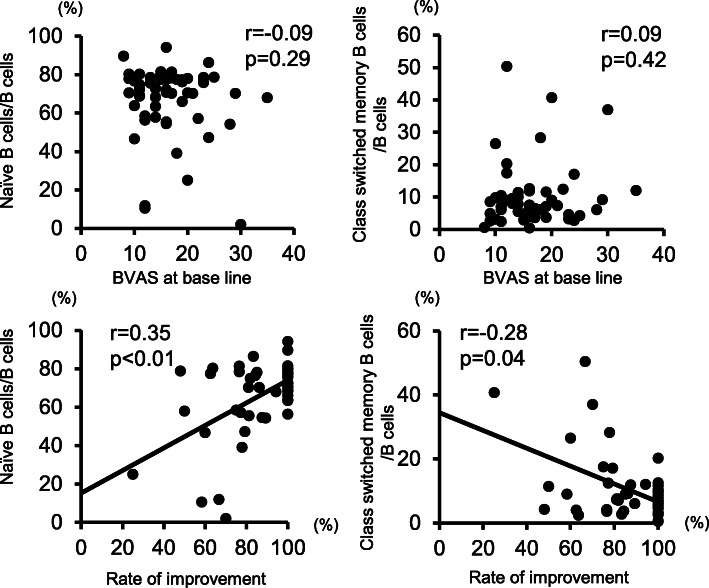
Correlations between clinical features and the proportion of peripheral naive B cells or class-switched B cells at baseline. Correlations between disease activity at baseline and the proportion of peripheral naive B cells or class-switched B cells at baseline (*top*). Correlations between the rate of improvement in BVAS and the proportion of peripheral naive B cells or class-switched B cells at baseline (*bottom*). The correlation between immune cell phenotypes and clinical features was analyzed by the Pearson product-moment correlation coefficient. The level of significance was considered *p* < 0.05. *r*, correlation coefficient

Next, the association between peripheral CD4^+^ T and B cell phenotypes and clinical signs before treatment initiation was analyzed (Fig. 3, Supplementary Table S3). Proportions of peripheral T and B cell phenotypes did not correlate with BVAS at baseline. However, among peripheral B cells, the proportion of naive B cells positively correlated with the rate of improvement in BVAS at 6 months after treatment initiation (*r* = 0.35, *p* < 0.01), whereas the proportion of class-switched memory B cells negatively correlated with this rate (*r* = − 0.28, *p* = 0.04). There was no correlation between the proportion of plasmablasts and the rate of BVAS improvement (Supplementary Fig. 5A).

### Association of excessive B cell differentiation with treatment resistance

**Fig. 4:**
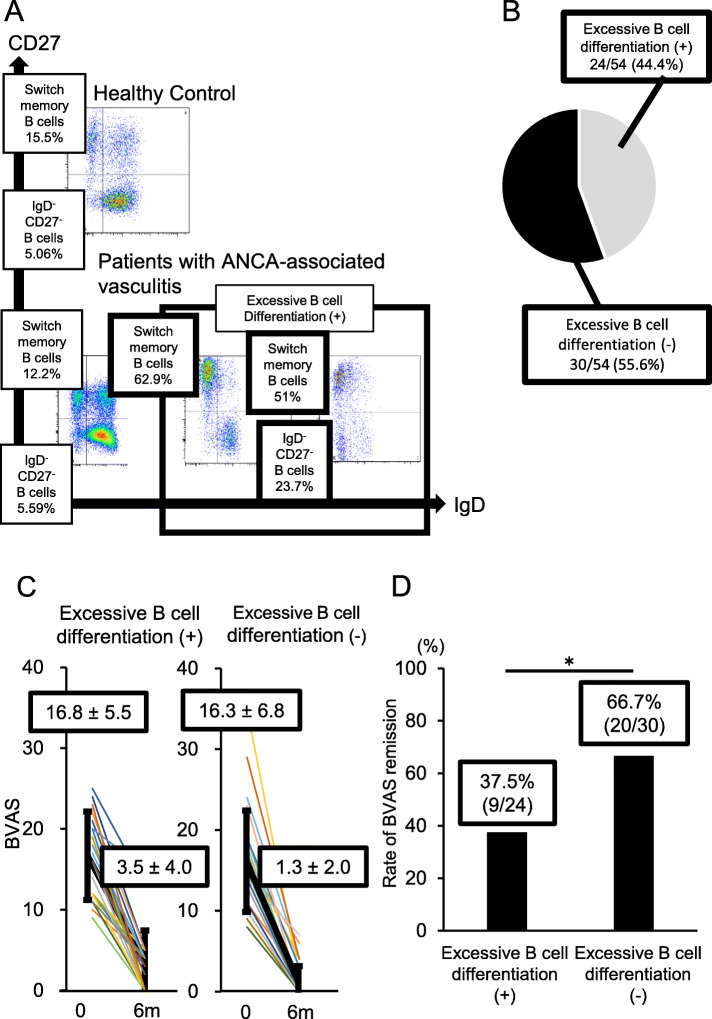
Identification and proportion of excessive B cell differentiation. **a** Human peripheral blood CD19+CD20+ B cells were stained with CD27 and IgD and separated into four phenotypes: CD27^−^IgD^−^, IgM+ unswitched memory (CD27^+^IgD^+^), class-switched memory (CD27^+^IgD^−^), and naive (CD27^−^IgD^+^) B cells, as indicated by the four quadrants. Dot plots show representative data from healthy control subjects (*n* = 15, *top*), AAV patients without excessive B cell differentiation (*bottom left*), and AAV patients with excessive B cell differentiation (*bottom middle and right*). **b** Proportions of AAV patients with (*grey*) and without (*black*) excessive B cell differentiation. **c** Changes in BVAS after 6 months of induction therapy in patients with and without excessive B cell differentiation. Data are mean ± SD of BVAS in each group. *p < 0.01 according to paired Student’s *t* test. **d** Rates of remission in BVAS in patients with and without excessive B cell differentiation. Data show the median rate of improvement in BVAS in each group. *p < 0.01 according to Fisher’s exact test

Next, among the AAV patients, we compared the response to treatment between patients with and without excessive B cell differentiation. Patients with excessive B cell differentiation were defined as those in whom the proportion of class-switched memory B cells or IgD^−^CD^−^ B cells among all B cells (CD19^+^CD20^+^) was > 2 SDs higher than the mean in the HCs (class-switched memory B cells/B cells > 23% or IgD^−^CD27^−^ B cells/B cells > 11.3%) (Fig. 4a). Based on this definition, 24 of the 54 AAV patients (44.4%) showed excessive B cell differentiation (Fig. 4b).

Between the patients with and without excessive B cell differentiation, no notable differences were observed concerning patient characteristics or T cell phenotypes before administration of remission induction therapy (Supplementary Tables S4 and S5). However, after 6 months therapy, BVAS was significantly higher in patients with excessive B cell differentiation than in those without (*p* < 0.01, Fig. 4c), and the rate of remission in BVAS was significantly lower in those with excessive B cell differentiation at 6 months [presence of excessive B cell differentiation = 37.5%, absence of excessive B cell differentiation = 66.7%, *p* < 0.01] (Fig. 4d). On the other hand, the rate of BVAS remission at 6 months was compared between a group of patients with AAV (*n* = 39) with a proportion of plasmablasts ≥ HC mean + 2SD (plasmablasts/CD19^+^ cells ≥ 0.6%) and another group (*n* = 15) with the same parameter < HC mean + 2SD. This comparison revealed no significant inter-group difference (48.7%:66.7%, *p* = 0.36) (Supplementary Fig. 5B).

MPA is more frequently therapy-resistant than is GPA, and it is possible that in the present study, the higher percentage of MPA cases in the CY-treated group (although this difference was not statistically significantly) affected the results. However, among patients with MPA free of excessive B cell differentiation, the rate of BVAS remission did not differ between the CY-treated group (75%, *n* = 8) and the RTX-treated group (55%, *n* = 11) (*p* = 0.63). On the other hand, among patients with MPA showing excessive B cell differentiation, the rate of BVAS remission was significantly lower in the CY-treated group (0%, *n* = 7) than in the RTX-treated group (75%, *n* = 8) (*p* < 0.01). Few patients with GPA were studied, and there was no significant difference between the rates of BVAS remission among those patients in the two treatment groups. These results, shown in Supplementary Fig. 4 (A, B), suggest that excessive B cell differentiation is probably involved more closely in the resistance to treatment than the type of vasculitis. These results reveal that patients with excessive B cell differentiation are resistant to treatment.

### Favorable effectiveness of RTX in improving BVAS, reducing GC use, and increasing survival among patients with circulating B cell abnormalities

**Fig. 5:**
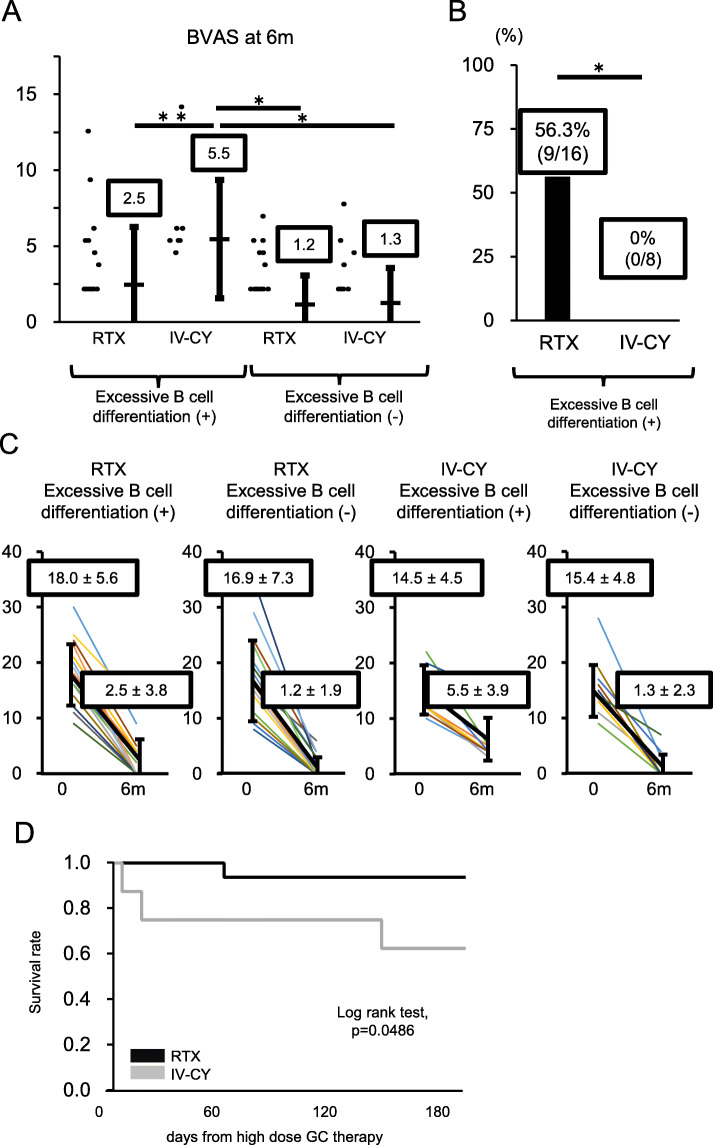
RTX was effective to conventional immunosuppressants in patients with excessive B cell differentiation. **a** Rate of improvement in BVAS after 6 months of induction therapy in patients with and without excessive B cell differentiation by treatment group. Data show the median rate of improvement in BVAS. **p < 0.05, *p < 0.01 according to Bonferroni method. **b** Rate of remission after 6 months of induction therapy in patients with excessive B cell differentiation by treatment group. *p < 0.01 according to Fisher’s exact test. **c** Changes in BVAS after 6 months of induction therapy in patients with and without excessive B cell differentiation by treatment group. Data are mean ± SD of BVAS in each group. **d** Differences between RTX and IV-CY groups in survival rates after 6 months of induction therapy in patients with excessive B cell differentiation

Lastly, the two treatment groups were further divided according to the presence or absence of excessive B cell differentiation, and responses to treatment were compared among the four resulting treatment groups. No significant differences were observed in disease duration, age, type of vasculitis, BVAS, or GC dose (Table 3). After 6 months of remission induction therapy, patients with excessive B cell differentiation in the IV-CY group had significantly higher BVAS than patients in the other three groups (Fig. 5a, c). Meanwhile, patients with excessive B cell differentiation in the RTX group had significantly lower BVAS at 6 months after treatment initiation compared with those with excessive B cell differentiation in the IV-CY group. BVAS in patients with excessive B cell differentiation in the RTX group were not significantly different from those in patients without excessive B cell differentiation, in either the RTX or IV-CY groups (Fig. 5a, c).

**Table 3:** Baseline characteristics of AAV patients with and without excessive B cell differentiation by treatment group

Variables	Rituximab group, *n* = 34		IV-CY group, *n* = 20		*p* value
Excessive B cell differentiation	+	−	+	−	
*n* (%)	16 (24.1%)	18 (27.6%)	8 (14.8%)	12 (22.2%)	
Age (years)	71.6 (8.3)	69.6 (9.5)	69.4 (8.6)	71.7 (7.8)	0.83
Gender, *n* (% female)	8 (57.1%)	11 (68.8%)	4 (44.4%)	8 (42.1%)	0.67
Disease duration (month)	2 (2–4)	3 (1–7)	3 (2–7)	4 (1–6)	0.70
New onset, *n* (%)	16 (100.0%)	18 (100.0%)	8 (100.0%)	12 (100.0%)	1.00
ANCA-positive at diagnosis, *n* (%)					
Proteinase 3 ANCA	3 (18.8%)	1 (5.6%)	0 (0.0%)	0 (0.0%)	0.15
Myeloperoxidase-ANCA	11 (68.8%)	15 (83.3%)	7 (87.5%)	11 (91.7%)	0.43
Proteinase 3 + myeloperoxidase-ANCA	1 (6.3%)	2 (11.1%)	1 (12.5%)	0 (0.0%)	0.65
ANCA-associated vasculitis type, *n* (%)					0.35
MPA	8 (50.0%)	11 (61.1%)	7 (87.5%)	8 (66.7%)	
GPA	8 (50.0%)	7 (38.9%)	1 (12.5%)	4 (33.3%)	
BVAS	18.0 (5.6)	16.9 (7.3)	14.5 (4.5)	15.4 (4.8)	0.50
GC dose at baseline	54.6 (11.0)	57.7 (12.1)	62.5 (22.4)	61.0 (12.8)	0.51

Values listed as mean (SD) and median (minimum-maximum) unless otherwise stated. The statistical difference was determined by Bonferroni method, with chi-square test used for nominal variables. Difference with *p* < 0.05 was considered significant

Furthermore, patients with excessive B cell differentiation who achieved BVAS remission were compared. Although all patients receiving RTX achieved BVAS remission, none of those receiving IV-CY did. There were significantly more patients responding to treatment in the RTX group (RTX group = 56.3%, IV-CY group = 0.0%, *p* < 0.01) (Fig. 5b).

Next, in patients with excessive B cell differentiation, rates of GC dose reduction were compared. The RTX group showed higher rates of dose reduction at 3 months [RTX group = 67.6 ± 13.1%, IV-CY group = 47.2 ± 21.6%, *p* < 0.01] and 6 months [RTX group = 84.3 ± 7.0%, IV-CY group = 69.1 ± 21.9%, *p* = 0.03] of remission induction therapy than the IV-CY group (Supplementary Fig. 6).

Regarding the incidence rates of adverse events in patients with excessive B cell differentiation, no significant difference was observed between the RTX and IV-CY groups. However, the incidence of severe infection was lower in the RTX group (RTX group = 4/16 patients, conventional therapy group = 6/8 patients, *p* = 0.03, Fisher’s exact test). Moreover, the 6-month survival rate after administration of high-dose GC therapy was significantly higher in the RTX group than in the IV-CY group (RTX group = 93.8%, IV-CY group = 62.5%, *p* = 0.0486) (Fig. 5d).

## Discussion

In the present study, which included patients with highly active AAV who were admitted to our hospital, we analyzed phenotypes of T and B cells before remission induction therapy to assess the clinical significance of these phenotypes and to differentiate the use of RTX and CY according to abnormal cell differentiation. Based on phenotyping of peripheral lymphocytes in this study, AAV patients exhibited a lower proportion of IgM+ unswitched memory B cells and a higher proportion of IgD^−^CD27^−^ B cells. Thus, B cells were more differentiated in many patients with highly active AAV compared to those in HCs.

Regarding the association between peripheral B cells and clinical findings, it has been reported that peripheral CD5^+^ B cells are associated with relapse after remission induction therapy with RTX [10, 11]. However, because there has been no study comparing abnormal differentiation and clinical findings, it has remained unknown which lymphocyte phenotype is associated with prognosis. The present study revealed that B cell phenotypes were not associated with disease activity or clinical findings but were correlated with responses to treatment. As the number of highly differentiated B cells increased, patients were more resistant to treatment. However, when patients with increased class-switched memory B cells or IgD^−^CD27^−^ B cells, defined as those with excessive B cell differentiation, were compared, no differences were observed in either baseline patient characteristics or T cell phenotypes. The presence of abnormal B cell phenotypes could not be predicted from ANCA levels, other baseline patient characteristics, or disease activity. In contrast, many patients with excessive B cell differentiation were resistant to treatment so that detection of excessive B cell differentiation may be important for predicting prognosis.

The above indicates that changes in prognosis mainly based on excessive B cell differentiation may be useful for differentiating between the use of RTX, which is used for B cell depletion therapy, and CY. The present study revealed that, in patients with excessive B cell differentiation, the concomitant use of RTX was more effective than that of conventional therapy and yielded improvements similar to that achieved by patients without excessive B cell differentiation. In addition to showing that RTX is effective in patients who are resistant to CY or experience relapse after treatment with CY [12–15], various clinical studies have demonstrated that RTX is as effective as CY against AAV accompanied by serious organ involvement [3, 16]. However, it is still unknown which patients should be treated with RTX and which with CY. The present study suggests that RTX is better indicated for patients with abnormal B cells. Moreover, this study also showed that the incidence of adverse events was lower in patients with excessive B cell differentiation who received a combination of RTX and GC than in those treated with concomitant conventional therapy, as GC doses could be reduced in the early stages. This may improve the survival rate in these patients.

Although the characteristics of B cells in AAV patients have been described in many reports, the importance of B cells remains controversial. Culton et al. [17] and Tadema et al. [18] reported that, compared with healthy individuals, AAV patients have more naive B cells (CD19^+^CD27^−^CD38^−/low^) and fewer memory B cells (CD19^+^CD27^+^CD38^−/low^). In contrast, Culton et al. [17] reported that, compared with healthy individuals, AAV patients have more CD19^hi^ memory B cells, which have an elevated capacity to produce autoantibodies. Moreover, Lepse et al. [19] reported that the proportion of regulatory B cells decreases in patients with highly active AAV. In the present study, phenotyping of peripheral lymphocytes revealed that AAV patients exhibited a low proportion of IgM+ unswitched memory B cells and a high proportion of IgD^−^CD27^−^ B cells. Thus, in many patients with highly active AAV, B cells were more differentiated than in HCs, similar to a trend described in previous reports [17, 18]. To date, several reports have been published concerning the relationship between peripheral blood B cells and responses of autoimmune disease to treatment. Lanzillotta et al. reported that in patients with IgG4-related disease, an increase in peripheral blood memory B cell count (CD19^+^CD20^+^CD27^+^CD38^−^ cells) 6 months after the beginning of glucocorticoid treatment is associated with relapse [20]. Yusof et al. reported that among RTX-treated patients with AAV, the length of time until relapse was longer in cases showing re-proliferation of peripheral blood naïve B cells (CD19^+^CD27^−^ cells) rather than peripheral blood memory B cells (CD19^+^CD27^+^ B cells) 6 months after the beginning of RTX therapy for removal of peripheral blood B cells [21]. These previous findings suggest the possibility that memory B cells, including class-switched memory B cells, are associated with relapse and resistance to treatment. These findings do not contradict the results of the present study.

The present study has several limitations. The RTX and IV-CY groups were not matched for baseline patient characteristics, and they were not compared in a double-blind manner. Thus, it cannot be ruled out that the selection of treatment was biased. However, no significant differences were observed between the RTX and IV-CY groups in terms of baseline patient characteristics, such as age, sex, disease duration, and disease activity, and the groups were compared for efficacy under the same conditions. The data from this study are preliminary in nature, because the number of cases with excessive B cell differentiation was small. Further studies on large number of patients are needed to confirm the utility of the reported findings.

## Conclusions

We performed 8-color flow cytometry in AAV patients before the administration of remission induction therapy and analyzed whether they exhibited excessive B cell differentiation. In patients with a high proportion of class-switched memory B cells or IgD^−^CD27^−^ B cells, GC therapy combined with RTX is expected to be more effective than conventional therapy, allowing for early reduction of GC doses and yielding a higher survival rate. These results provide insight for the development of subclassifications of AAV based on analysis of immunophenotypes by flow cytometry and of corresponding therapeutic interventions in the form of precision medicine.

## Supplementary information

**Additional file 1 Supplementary Fig. 1**. Identification of T and B cell phenotypes by 8-color antibody staining. **Supplementary Fig. 2**. Comparison of the actual number of peripheral blood IgM unswitched memory B cells and IgG-CD27- B cells between healthy controls and patients with ANCA-related vasculitis. **Supplementary Fig. 3**. Correlations between the proportion of peripheral class switched memory B cells and IgD-CD27- B cells at baseline. **Supplementary Fig. 4**. Comparison between rates of BVAS improvement 6 months after the beginning of remission induction therapy in the RTX and IV-CY groups according to disease type (MPA and GPA) and presence/absence of excessive B cell differentiation. **Supplementary Fig. 5**. Association between plasmablasts and resistance to treatment. **Supplementary Fig. 6**. Changes in the rate of glucocorticoid reduction in patients with and without excessive B cell differentiation by treatment group. **Supplementary Table S1**. Eight-color antibody panels used in the study. **Supplementary Table S2**. Differences in the proportions of circulating T cell and B cell phenotypes between patients with AAV at baseline and sex-matched healthy control subjects. **Supplementary Table S3**. Correlation between disease activity at baseline or rate of improvement in BVAS and the proportion of circulating T cell and B cell phenotypes. **Supplementary Table S4**. Baseline characteristics of AAV patients with and without excessive B cell differentiation. **Supplementary Table S5**. Differences in proportions of circulating T cells between AAV patients with and without excessive B cell differentiation.

## Data Availability

The datasets used and/or analyzed during the current study are available from the corresponding author on reasonable request.

## Abbreviations

AAV: Anti-neutrophil cytoplasmic autoantibody associated vasculitis
ANCA: Anti-neutrophil cytoplasmic autoantibody
AZA: Azathioprine
BVAS: Birmingham Vasculitis Activity Score
CD: Cluster of differentiation
GC: Glucocorticoids
HC: Healthy controls
Ig: Immunoglobulin
IV-CY: Intravenous cyclophosphamide pulse
PBMC: Peripheral blood mononuclear cells
RTX: Rituximab
SD: Standard deviation
