# Supplementary material for: Favorable efficacy of rituximab in ANCA-associated vasculitis patients with excessive B cell differentiation
Source: Arthritis Res Ther. 2020 Jun 15;22:141. doi: 10.1186/s13075-020-02215-x (PMC7294638; doi:10.1186/s13075-020-02215-x)
Supplement: Supplementary file 1 — Additional file 1 Supplementary Fig. 1. Identification of T and B cell phenotypes by 8-color antibody staining. Supplementary Fig. 2. Comparison of the actual number of peripheral blood IgM unswitched memory B cells and IgG-CD27- B cells between healthy controls and patients with ANCA-related vasculitis. Supplementary Fig. 3. Correlations between the proportion of peripheral class switched memory B cells and IgD-CD27- B cells at baseline. Supplementary Fig. 4. Comparison between rates of BVAS improvement 6 months after the beginning of remission induction therapy in the RTX and IV-CY groups according to disease type (MPA and GPA) and presence/absence of excessive B cell differentiation. Supplementary Fig. 5. Association between plasmablasts and resistance to treatment. Supplementary Fig. 6. Changes in the rate of glucocorticoid reduction in patients with and without excessive B cell differentiation by treatment group. Supplementary Table S1. Eight-color antibody panels used in the study. Supplementary Table S2. Differences in the proportions of circulating T cell and B cell phenotypes between patients with AAV at baseline and sex-matched healthy control subjects. Supplementary Table S3. Correlation between disease activity at baseline or rate of improvement in BVAS and the proportion of circulating T cell and B cell phenotypes. Supplementary Table S4. Baseline characteristics of AAV patients with and without excessive B cell differentiation. Supplementary Table S5. Differences in proportions of circulating T cells between AAV patients with and without excessive B cell differentiation. [file 13075_2020_2215_MOESM1_ESM.zip › AAV bcell (AR&T) supplementary figure.pptx]

## Slide 1
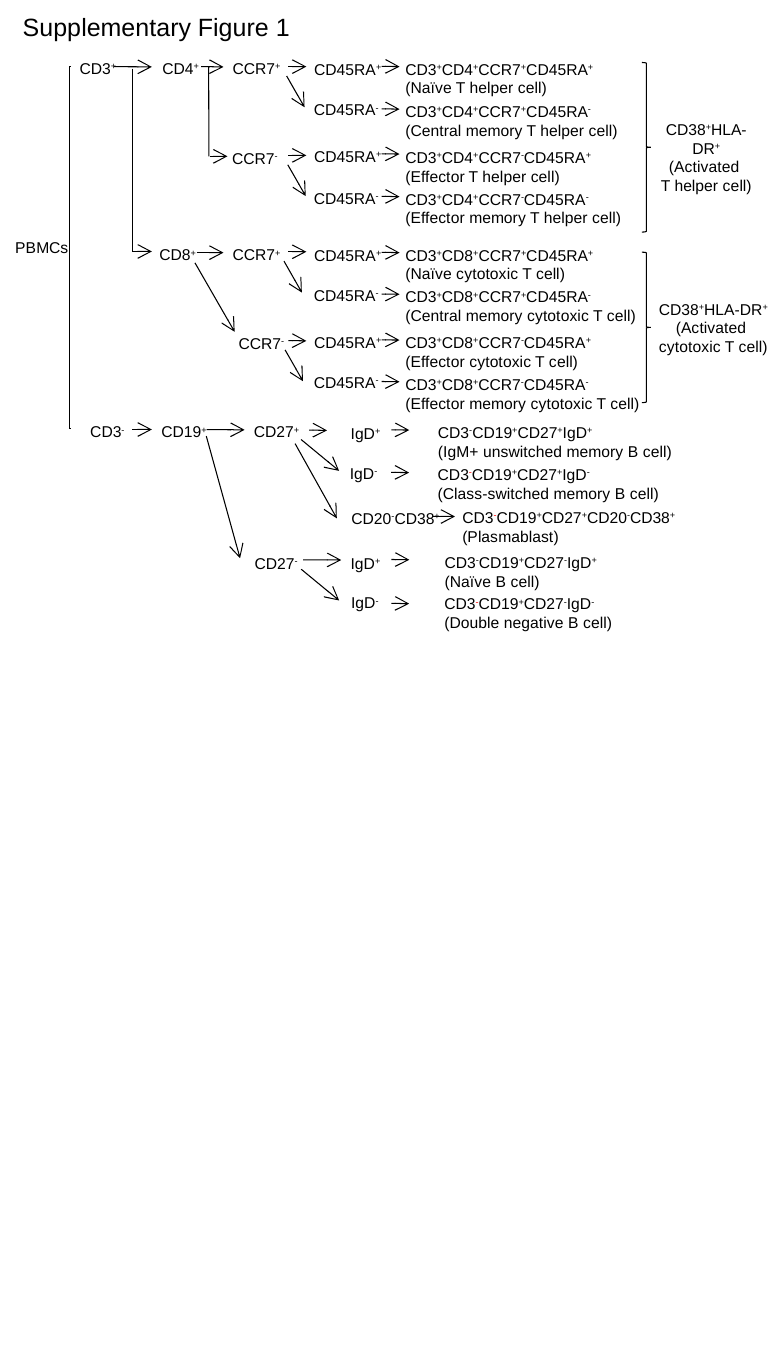

Supplementary Figure 1
CD3+
CD4+
CCR7+
CD45RA+
CD3+CD4+CCR7+CD45RA+
(Naïve T helper cell)
CD45RA-
CD3+CD4+CCR7+CD45RA-
(Central memory T helper cell)
CD38+HLA-DR+
(Activated
T helper cell)
CD45RA+
CD3+CD4+CCR7-CD45RA+
(Effector T helper cell)
CCR7-
CD45RA-
CD3+CD4+CCR7-CD45RA-
(Effector memory T helper cell)
PBMCs
CD8+
CCR7+
CD45RA+
CD3+CD8+CCR7+CD45RA+
(Naïve cytotoxic T cell)
CD45RA-
CD3+CD8+CCR7+CD45RA-
(Central memory cytotoxic T cell)
CD38+HLA-DR+
(Activated
cytotoxic T cell)
CD45RA+
CD3+CD8+CCR7-CD45RA+
(Effector cytotoxic T cell)
CCR7-
CD45RA-
CD3+CD8+CCR7-CD45RA-
(Effector memory cytotoxic T cell)
CD3-
CD19+
CD27+
CD3-CD19+CD27+IgD+
(IgM+ unswitched memory B cell)
IgD+
IgD-
CD3-CD19+CD27+IgD-
(Class-switched memory B cell)
CD3-CD19+CD27+CD20-CD38+
(Plasmablast)
CD20-CD38+
CD3-CD19+CD27-IgD+
(Naïve B cell)
CD27-
IgD+
IgD-
CD3-CD19+CD27-IgD-
(Double negative B cell)

## Slide 2
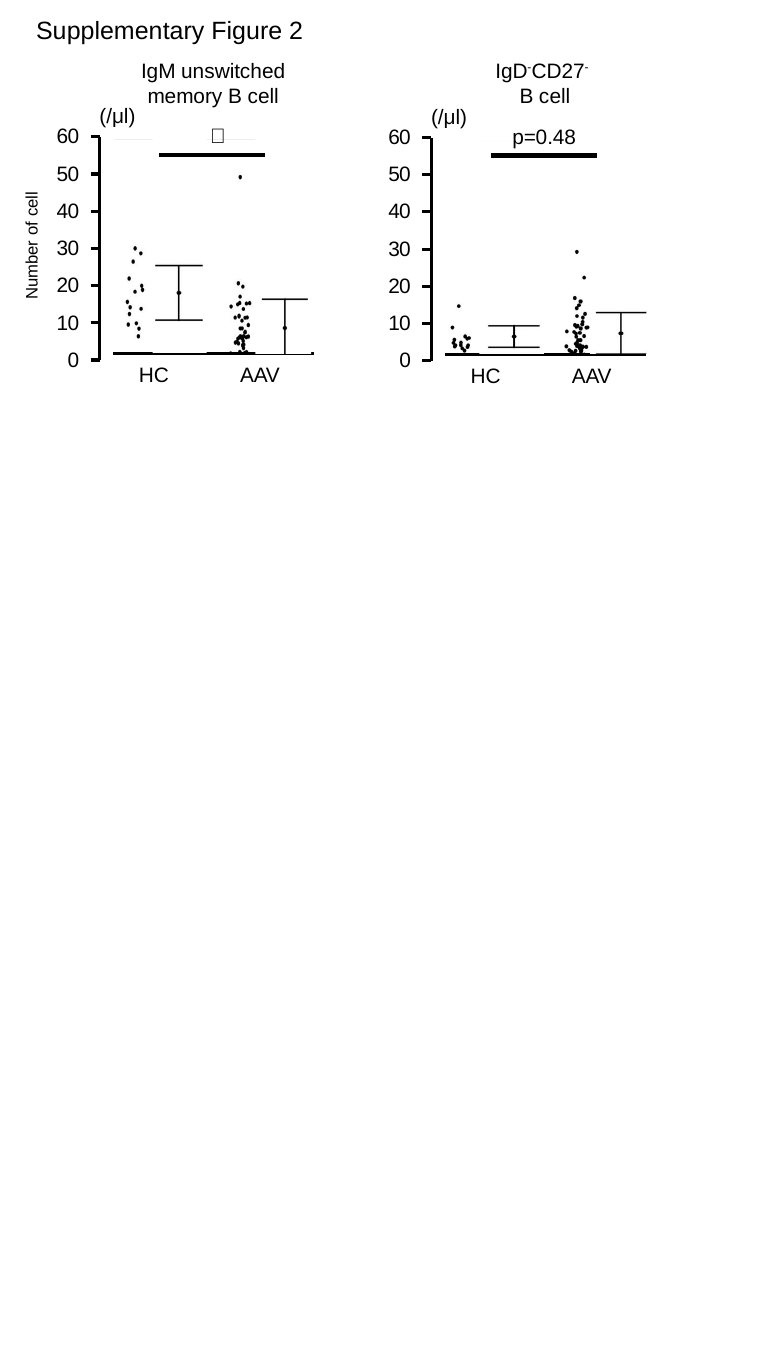

Supplementary Figure 2
IgM unswitched memory B cell
IgD-CD27-
B cell
(/μl)
(/μl)
＊
p=0.48
### Chart
| Category | |
|---|---|
### Chart
| Category | |
|---|---|
Number of cell
HC
AAV
HC
AAV

## Slide 3
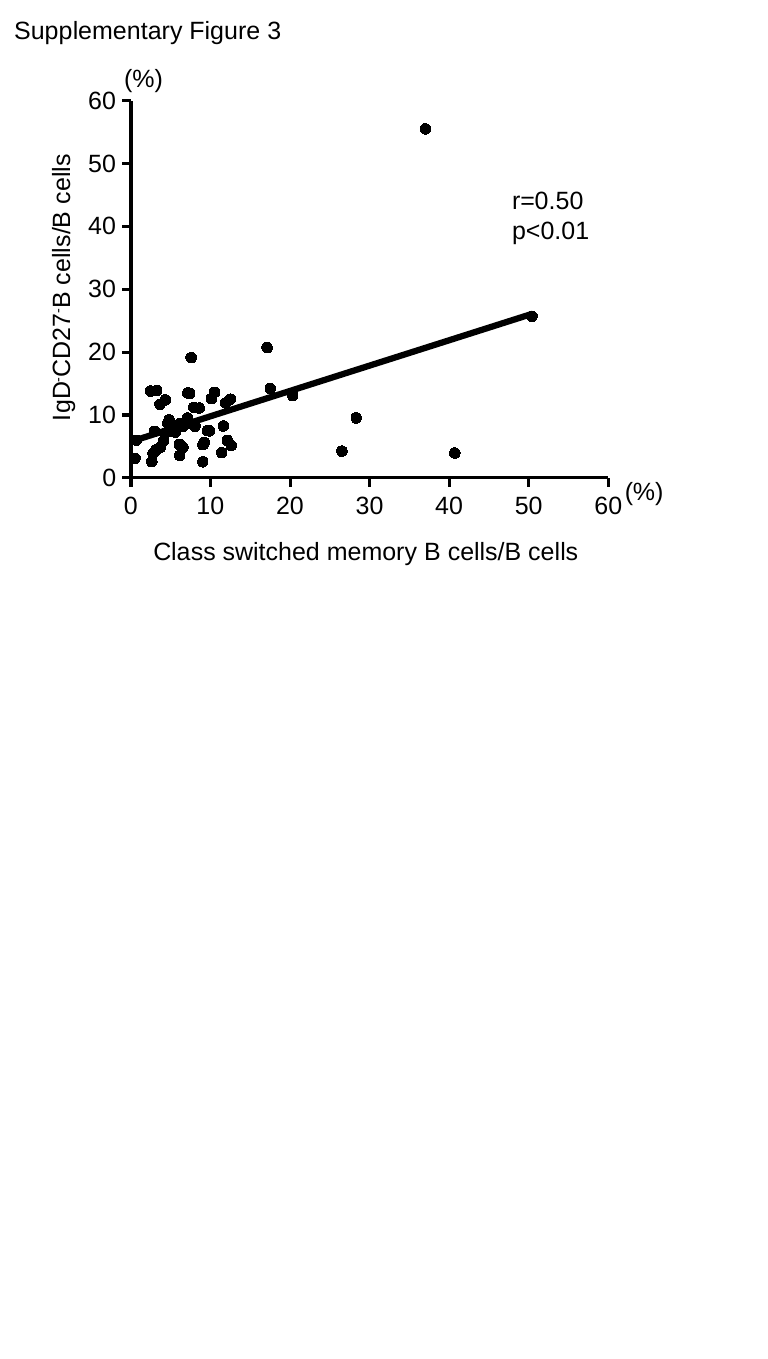

Supplementary Figure 3
(%)
### Chart
| Category | |
|---|---|r=0.50
p<0.01
IgD-CD27-B cells/B cells
(%)
Class switched memory B cells/B cells

## Slide 4
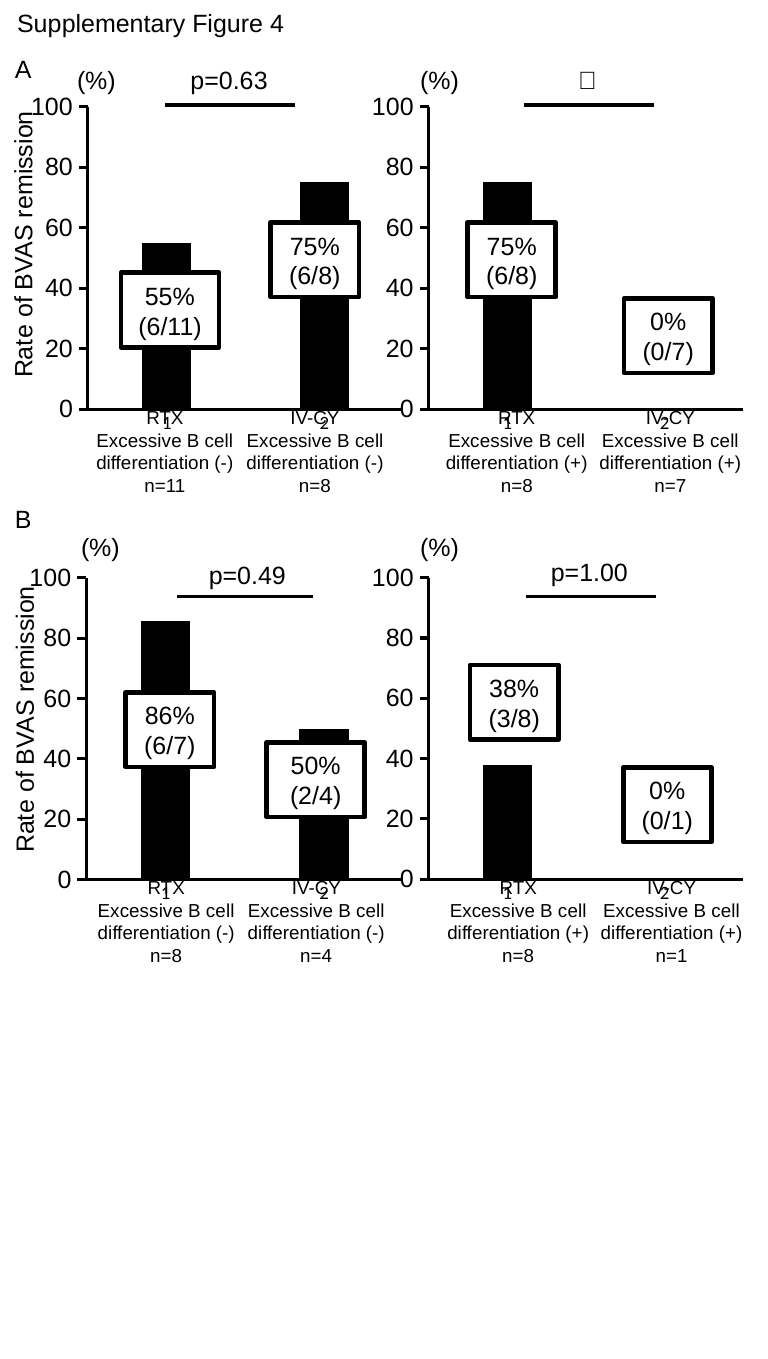

Supplementary Figure 4
A
(%)
p=0.63
(%)
＊
### Chart
| Category | |
|---|---|
### Chart
| Category | |
|---|---|Rate of BVAS remission
75%
(6/8)
75%
(6/8)
55%
(6/11)
0%
(0/7)
RTX
Excessive B cell
differentiation (-)
n=11
IV-CY
Excessive B cell
differentiation (-)
n=8
RTX
Excessive B cell
differentiation (+)
n=8
IV-CY
Excessive B cell
differentiation (+)
n=7
B
(%)
(%)
p=1.00
p=0.49
### Chart
| Category | |
|---|---|
### Chart
| Category | |
|---|---|38%
(3/8)
86%
(6/7)
Rate of BVAS remission
50%
(2/4)
0%
(0/1)
RTX
Excessive B cell
differentiation (-)
n=8
IV-CY
Excessive B cell
differentiation (-)
n=4
RTX
Excessive B cell
differentiation (+)
n=8
IV-CY
Excessive B cell
differentiation (+)
n=1

## Slide 5
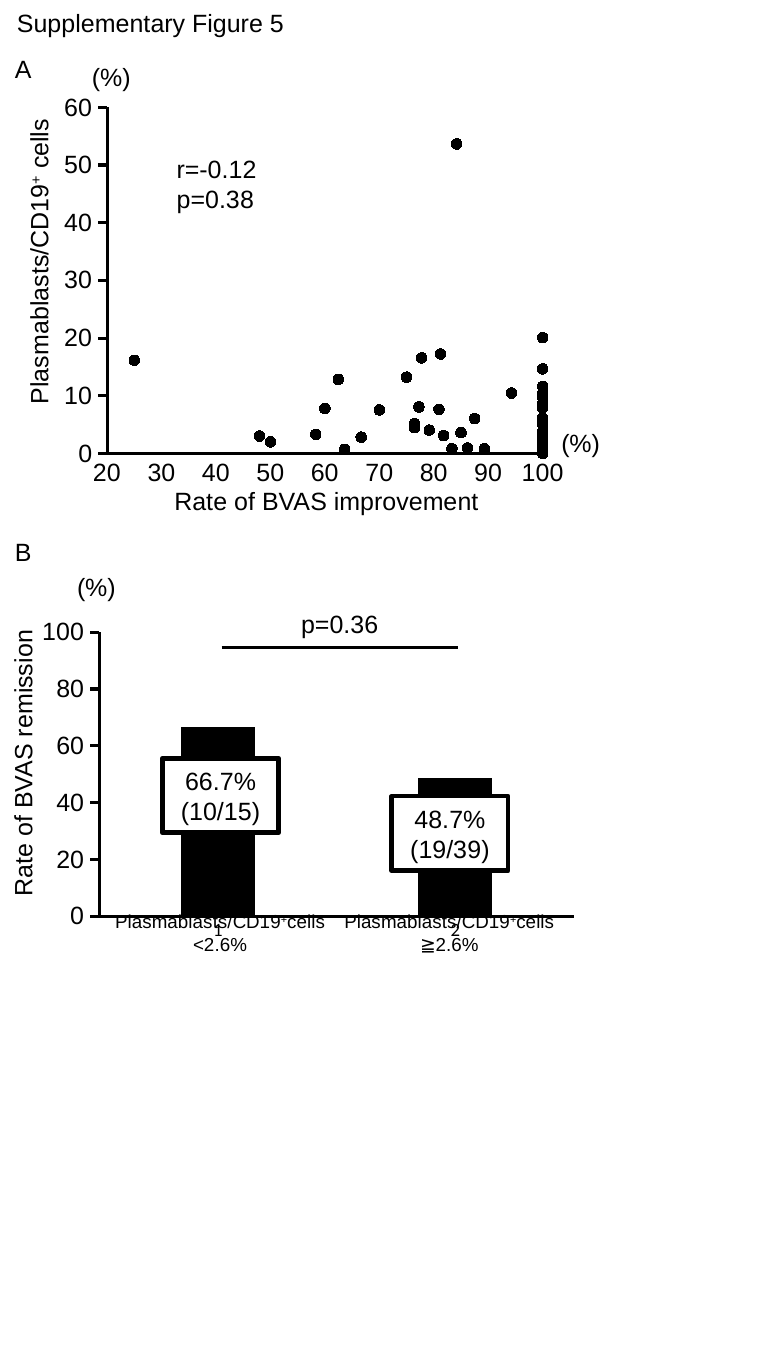

Supplementary Figure 5
A
(%)
### Chart
| Category | |
|---|---|r=-0.12
p=0.38
Plasmablasts/CD19+ cells
(%)
Rate of BVAS improvement
B
(%)
p=0.36
### Chart
| Category | |
|---|---|Rate of BVAS remission
66.7%
(10/15)
48.7%
(19/39)
Plasmablasts/CD19+cells
<2.6%
Plasmablasts/CD19+cells
≧2.6%

## Slide 6
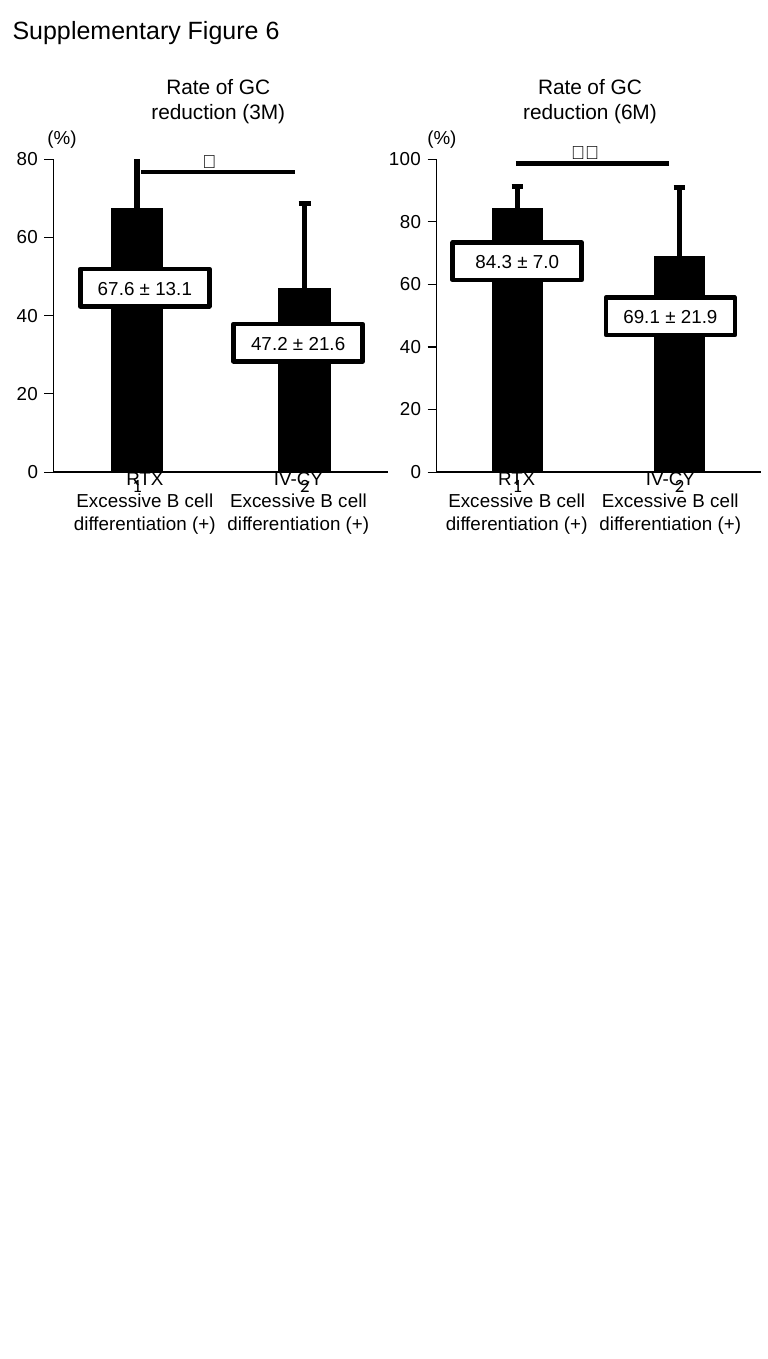

Supplementary Figure 6
Rate of GC reduction (3M)
Rate of GC reduction (6M)
(%)
(%)
＊＊
### Chart
| Category | |
|---|---|
### Chart
| Category | |
|---|---|＊
84.3 ± 7.0
67.6 ± 13.1
69.1 ± 21.9
47.2 ± 21.6
RTX
Excessive B cell
differentiation (+)
IV-CY
Excessive B cell
differentiation (+)
RTX
Excessive B cell
differentiation (+)
IV-CY
Excessive B cell
differentiation (+)
