# Supplementary material for: Favorable efficacy of rituximab in ANCA-associated vasculitis patients with excessive B cell differentiation
Source: Arthritis Res Ther. 2020 Jun 15;22:141. doi: 10.1186/s13075-020-02215-x (PMC7294638; doi:10.1186/s13075-020-02215-x)
Supplement: Supplementary file 1 — Additional file 1 Supplementary Fig. 1. Identification of T and B cell phenotypes by 8-color antibody staining. Supplementary Fig. 2. Comparison of the actual number of peripheral blood IgM unswitched memory B cells and IgG-CD27- B cells between healthy controls and patients with ANCA-related vasculitis. Supplementary Fig. 3. Correlations between the proportion of peripheral class switched memory B cells and IgD-CD27- B cells at baseline. Supplementary Fig. 4. Comparison between rates of BVAS improvement 6 months after the beginning of remission induction therapy in the RTX and IV-CY groups according to disease type (MPA and GPA) and presence/absence of excessive B cell differentiation. Supplementary Fig. 5. Association between plasmablasts and resistance to treatment. Supplementary Fig. 6. Changes in the rate of glucocorticoid reduction in patients with and without excessive B cell differentiation by treatment group. Supplementary Table S1. Eight-color antibody panels used in the study. Supplementary Table S2. Differences in the proportions of circulating T cell and B cell phenotypes between patients with AAV at baseline and sex-matched healthy control subjects. Supplementary Table S3. Correlation between disease activity at baseline or rate of improvement in BVAS and the proportion of circulating T cell and B cell phenotypes. Supplementary Table S4. Baseline characteristics of AAV patients with and without excessive B cell differentiation. Supplementary Table S5. Differences in proportions of circulating T cells between AAV patients with and without excessive B cell differentiation. [file 13075_2020_2215_MOESM1_ESM.zip › AAV bcell (AR&T) Supplementary_materials.docx]

**Supplementary Figure 1. Identification of T and B cell phenotypes by 8‑color antibody staining**

**Supplementary Figure 2. Comparison of the actual number of peripheral blood IgM unswitched memory B cells and IgG-CD27- B cells between healthy controls and patients with ANCA-related vasculitis**

Left: Comparison of the actual number of IgM+ unswitched memory B cells between healthy controls and patients with ANCA-related vasculitis. Right: Comparison of the actual number of IgD-CD27- B cells between healthy controls and patients with ANCA-related vasculitis.

*p < 0.01 according to Student's t-test.

**Supplementary Figure 3. Correlations between the proportion of peripheral class switched memory B cells and IgD^-^CD27^-^ B cells at baseline**

The correlation between the proportion of peripheral class-switched memory B cells and IgD^-^CD27^-^ B cells at baseline was analyzed by the Pearson product-moment correlation coefficient. p < 0.05 was considered as statistically significant. r: correlation coefficient.

**Supplementary Figure 4. Comparison between rates of BVAS improvement 6 months after the beginning of remission induction therapy in the RTX and IV-CY groups according to disease type (MPA and GPA) and presence/absence of excessive B cell differentiation**

(A) Comparison between rates of BVAS improvement 6 months after the beginning of remission induction therapy in the RTX and IV-CY groups among MPA patients without excessive B cell differentiation (left); comparison between rates of BVAS improvement 6 months after the beginning of remission induction therapy in the RTX and IV-CY groups among MPA patients showing excessive B cell differentiation.

(B) Comparison between rates of BVAS improvement 6 months after the beginning of remission induction therapy in the RTX and IV-CY groups among GPA patients without excessive B cell differentiation (left); comparison between rates of BVAS improvement 6 months after the beginning of remission induction therapy in the RTX and IV-CY groups among GPA patients showing excessive B cell differentiation. **p* < 0.01 according to Fisher's exact test.

**Supplementary Figure 5. Association between plasmablasts and resistance to treatment**

(A) Correlations between rates of improvement in BVAS and the proportion of peripheral plasmablasts at baseline. The correlation between the rate of improvement in BVAS and plasmablasts was analyzed by the Pearson product-moment correlation coefficient. p < 0.05 was considered as statistically significant. r: correlation coefficient.

(B) Comparison between rates of BVAS improvement 6 months after the beginning of remission induction therapy in patients with a proportion of peripheral blood plasmablasts higher than the HC average + 2SD (2.6%) and patients with a proportion lower than 2.6%. P-value according to Fisher's exact test.

**Supplementary Figure 6. Changes in the rate of glucocorticoid reduction in patients with and without excessive B cell differentiation by treatment group**

Differences in the rate of glucocorticoid reduction after 3 months (*left*) and 6 months (*right*) of induction therapy in patients with excessive B cell differentiation between the RTX group and IV-CY groups. **p* < 0.01 according to Student’s t-test.

**Supplementary Table S1. Eight‑color antibody panels used in the study**

|  | T cells | B cells |
| --- | --- | --- |
| FITC | Live or dead | Live or dead |
|  |  |  |
| PE | CCR7 | CD24 |
|  |  |  |
| PerCP-Cy5.5 | CD4 | CD19 |
|  |  |  |
| PE-Cy7 | CD45RA | CD27 |
|  |  |  |
| APC | CD38 | CD38 |
|  |  |  |
| APC-H7 | CD8 | CD20 |
|  |  |  |
| V450 | CD3 | CD3 |
|  |  |  |
| V500 | HLA-DR | IgD |
|  |  |  |

**Supplementary Table S2. Differences in the proportions of circulating T cell and B cell phenotypes between patients with AAV at baseline and sex-matched healthy control subjects**

|  |  | Healthy control  n=15 | AAV patients  n=54 | p value |
| --- | --- | --- | --- | --- |
|  | Age (years) | 69.8 (3.7) | 70.6 (8.3) | 0.72 |
|  | Gender, n (% female) | 9 (60.0%) | 29 (53.7%) | 0.77 |
| CD4  T cells | Naive | 39.3 (16.0) | 50.6 (18.8) | 0.04 |
|  | Central memory | 36.9 (13.4) | 29.2 (11.8) | 0.03 |
|  | Effector memory | 19.0 (11.0) | 15.2 (9.9) | 0.20 |
|  | Effector | 4.7 (7.2) | 5.0 (7.4) | 0.91 |
|  | Activated | 7.3 (2.5) | 10.2 (7.5) | 0.14 |
| CD8  T cells | Naive | 24.6 (14.0) | 35.1 (18.9) | 0.07 |
|  | Central memory | 21.2 (16.4) | 21.2 (15.9) | 0.99 |
|  | Effector memory | 31.2 (19.4) | 24.4 (15.9) | 0.17 |
|  | Effector | 32.8 (19.3) | 25.8 (18.3) | 0.19 |
|  | Activated | 15.2 (7.8) | 21.3 (14.4) | 0.12 |
| B cells | Naive | 64.3 (16.1) | 66.6 (19.0) | 0.67 |
|  | IgM+ unswitched memory | 19.0 (6.9) | 12.1 (6.7) | <0.01 |
|  | Class-switched memory | 11.3 (8.3) | 10.1 (9.8) | 0.67 |
|  | Double negative | 5.4 (2.7) | 9.8 (7.9) | 0.04 |
|  | Plasmablasts | 2.1 (1.0-2.7) | 5.1(2.2–8.3) | 0.04 |

Values listed as mean (SD) and median (minimum-maximum) unless otherwise stated. Proportions of peripheral T and B cell phenotypes in healthy control subjects and AAV patients at baseline. The statistical difference was determined by Student’s t-test and Mann-Whitney's U test. Difference with p<0.05 was considered significant.

**Supplementary Table S3. Correlation between disease activity at baseline or rate of improvement in BVAS and the proportion of circulating T cell and B cell phenotypes**

|  |  |  | BVAS  at base line | Rate of improvement |
| --- | --- | --- | --- | --- |
| CD4 T cells | Naive | r | 0.20 | -0.07 |
|  |  | p value | 0.26 | 0.61 |
|  | Central memory | r | -0.13 | -0.05 |
|  |  | p value | 0.54 | 0.73 |
|  | Effector memory | r | -0.17 | 0.12 |
|  |  | p value | 0.54 | 0.38 |
|  | Effector | r | -0.22 | 0.06 |
|  |  | p value | 0.28 | 0.67 |
|  | Activated | r | 0.12 | -0.11 |
|  |  | p value | 0.86 | 0.46 |
| CD8 T cells | Naive | r | 0.03 | 0.01 |
|  |  | p value | 0.86 | 0.92 |
|  | Central memory | r | -0.03 | -0.23 |
|  |  | p value | 0.94 | -0.09 |
|  | Effector memory | r | 0.04 | 0.53 |
|  |  | p value | 0.89 | 0.81 |
|  | Effector | r | 0.03 | 0.04 |
|  |  | p value | 0.88 | 0.75 |
|  | Activated | r | 0.18 | -0.18 |
|  |  | p value | 0.20 | 0.19 |
| B cells | Naive | r | -0.09 | 0.35 |
|  |  | p value | 0.29 | <0.01 |
|  | IgM+ unswitched memory | r | 0.03 | -0.18 |
|  |  | p value | 0.56 | 0.18 |
|  | Class-switched memory | r | 0.09 | -0.28 |
|  |  | p value | 0.42 | 0.04 |
|  | Double negative | r | 0.11 | -0.10 |
|  |  | p value | 0.41 | 0.45 |
|  | Plasmablasts | r | 0.07 | -0.15 |
|  |  | p value | 0.67 | 0.29 |

The correlation between immune cell phenotypes and clinical features was analyzed by the Pearson product-moment correlation coefficient. The level of significance was considered p<0.05. r: correlation coefficient.

**Supplementary Table S4. Baseline characteristics of AAV patients with and without excessive B cell differentiation**

| Variables | Excessive B cell differentiation (+)  n=24 | Excessive B cell differentiation (-)  n=30 | p value |
| --- | --- | --- | --- |
| Age (years) | 71.0 (8.6) | 70.3 (8.2) | 0.78 |
| Gender, n (% female) | 15 (62.5%) | 14 (46.7%) | 0.28 |
| Disease duration (month) | 2 (2-4) | 3 (2-7) | 0.67 |
| ANCA-positive at diagnosis, n(%) |  |  |  |
| Proteinase 3 ANCA | 5 (20.8%) | 3 (10.0%) | 0.44 |
| Myeloperoxidase-ANCA | 20 (83.3%) | 28 (93.3%) | 0.39 |
| ANCA-associated vasculitis type, n(%) |  |  | 1.00 |
| MPA | 15 (62.5%) | 19 (63.3%) |  |
| GPA | 9 (37.5%) | 11 (36.7%) |  |
| BVAS | 16.8 (5.5) | 16.3 (6.3) | 0.76 |
| new onset, n(%) | 24 (100.0%) | 30 (100.0%) | 1.00 |
| GC dose at base line | 57.3 (15.7) | 59.0 (12.2) | 0.64 |
| Therapy, n |  |  | 0.78 |
| RTX | 16 | 18 |  |
| IV-CY | 8 | 12 |  |
| Organ involvement, n |  |  |  |
| Constitutional signs or symptoms | 24 | 30 | 1.00 |
| Cutaneous involvement | 0 | 2 | 0.50 |
| Mucous membranes and eyes | 3 | 1 | 0.65 |
| Ear, nose, and throat | 7 | 10 | 0.78 |
| Pulmonary involvement | 22 | 20 | 0.51 |
| Renal involvement | 18 | 28 | 0.12 |
| Neurologic involvement | 8 | 6 | 1.00 |

Values listed as mean (SD) and median (minimum-maximum) unless otherwise stated. Proportions of peripheral T and B cell phenotypes in healthy control subjects and AAV patients at baseline. The statistical difference was determined by Student’s t-test, Mann-Whitney's U test and . Difference with p<0.05 was considered significant.

**Supplementary Table S5.** **Differences in proportions of circulating T cells between AAV patients with and without excessive B cell differentiation**

|  |  | Excessive B cell differentiation (+)  n=24 | Excessive B cell differentiation (-)  n=30 | p value |
| --- | --- | --- | --- | --- |
| CD4 T cells | Naive | 53.5 (20.9) | 48.3 (16.9) | 0.32 |
|  | Central memory | 28.0 (12.6) | 30.1 (11.3) | 0.51 |
|  | Effector memory | 14.7 (11.1) | 15.6 (9.0) | 0.76 |
|  | Effector | 2.5 (1.6-4.3) | 2.4 (1-7-4.9) | 0.28 |
|  | Activated | 11.3 (9.9) | 9.4 (4.6) | 0.36 |
| CD8 T cells | Naive | 32.4 (18.9) | 37.2 (18.9) | 0.36 |
|  | Central memory | 21.0 (17.2) | 21.4 (15.2) | 0.92 |
|  | Effector memory | 20.7 (15.7) | 18.6 (14.2) | 0.60 |
|  | Effector | 23.4 (10.7-28.5) | 21.3 (15.3-33.9) | 0.75 |
|  | Activated | 22.3 (16.0) | 20.5 (13.2) | 0.65 |

Values listed as mean (SD) and median (minimum-maximum) unless otherwise stated. The statistical difference was determined by Student’s t-test and Mann-Whitney's U test. Difference with p<0.05 was considered significant.
